# Efficacy and safety of tisagenlecleucel in Japanese adult patients with relapsed/refractory diffuse large B-cell lymphoma

**DOI:** 10.1007/s10147-020-01699-6

**Published:** 2020-05-24

**Authors:** Hideki Goto, Shinichi Makita, Koji Kato, Kota Tokushige, Taizo Fujita, Koichi Akashi, Koji Izutsu, Takanori Teshima

**Affiliations:** 1 Department of Hematology, Graduate School of Medicine, Hokkaido University Faculty of Medicine, Sapporo, Japan; 2 Department of Hematology, National Cancer Center Hospital, Chuo-ku, Tokyo, Japan; 3 Department of Medicine and Biosystemic Science, Kyushu University Graduate School of Medical Sciences, Fukuoka, Japan; 4 Novartis Pharma K.K., Toranomon Minato-ku, Tokyo, Japan

**Keywords:** Tisagenlecleucel, CAR T-cell therapy, Diffuse large B-cell lymphoma, JULIET

## Abstract

**Background:**

Tisagenlecleucel demonstrated a high rate of durable response in adult patients with relapsed/refractory (r/r) diffuse large B-cell lymphoma (DLBCL) in the pivotal global phase 2 JULIET study. Here, we report the efficacy and safety of tisagenlecleucel in the Japanese subgroup.

**Methods:**

JULIET (NCT02445248) is a single-arm, open-label, multicenter, phase 2 study involving adult patients with r/r DLBCL who either relapsed after or were ineligible for autologous stem cell transplant. Primary endpoint was best overall response rate (ORR; complete response [CR] + partial response [PR]) as judged by an independent review committee.

**Results:**

In Japan, of 17 patients enrolled, 9 were infused with tisagenlecleucel and completed ≥ 3 months of follow-up. Best ORR was 77.8% (7/9; 95% confidence interval, 40.0–97.2), with 5 patients (55.6%) in CR and 2 (22.2%) in PR. Cytokine release syndrome (CRS) occurred in 6 patients (66.7%), with grade 3 CRS in 2 patients (Penn grading scale). Two patients received tocilizumab. Two deaths (22.2%) occurred more than 30 days after tisagenlecleucel infusion due to disease progression, neither of which were related to tisagenlecleucel.

**Conclusion:**

Tisagenlecleucel showed a high best ORR with a manageable safety profile, thus offering a new treatment option in selected Japanese patients with r/r DLBCL.

## Introduction

Diffuse large B-cell lymphoma (DLBCL) is an aggressive and the most common type of non-Hodgkin lymphoma (NHL) in Japan, with an incidence of 33% [1]. Survival rates have improved over time with immunochemotherapy combinations containing rituximab. However, nearly 40% of patients become relapsed/refractory (r/r) after treatment, are difficult to manage with conventional cytotoxic chemotherapy, and are associated with high treatment-related mortality rates [2]. In a retrospective study assessing the outcomes in 636 patients with refractory DLBCL, the overall response rate (ORR) was 26%, with only 7% of patients achieving a complete response (CR) and 18% achieving a partial response (PR), with a median overall survival (OS) of 6.3 months [3]. Despite overall improvements in outcomes of DLBCL, r/r disease remains a major cause of morbidity and mortality. To improve the outcome of r/r DLBCL, several novel therapies are being developed. Among them, anti-CD19 chimeric antigen receptor (CAR) T-cell therapy is considered one of the most promising and effective therapies for r/r DLBCL [4].

Tisagenlecleucel is a second-generation anti-CD19 CAR T-cell utilizing 4-1BB as a co-stimulatory domain. It targets CD19+ B cells and has shown efficacy against various subtypes of B-cell lymphomas, including DLBCL, in a single-center, phase 2a study [5]. Subsequently, a phase 2, multicenter, global, pivotal trial (JULIET; NCT02445248) was conducted, and tisagenlecleucel demonstrated a high rate of durable response in adults with r/r DLBCL [6]. Here, we report a subgroup analysis of the Japanese patients infused with tisagenlecleucel for r/r DLBCL in the JULIET trial.

## Patients and methods

### Study design and patient population

**Fig. 1:**
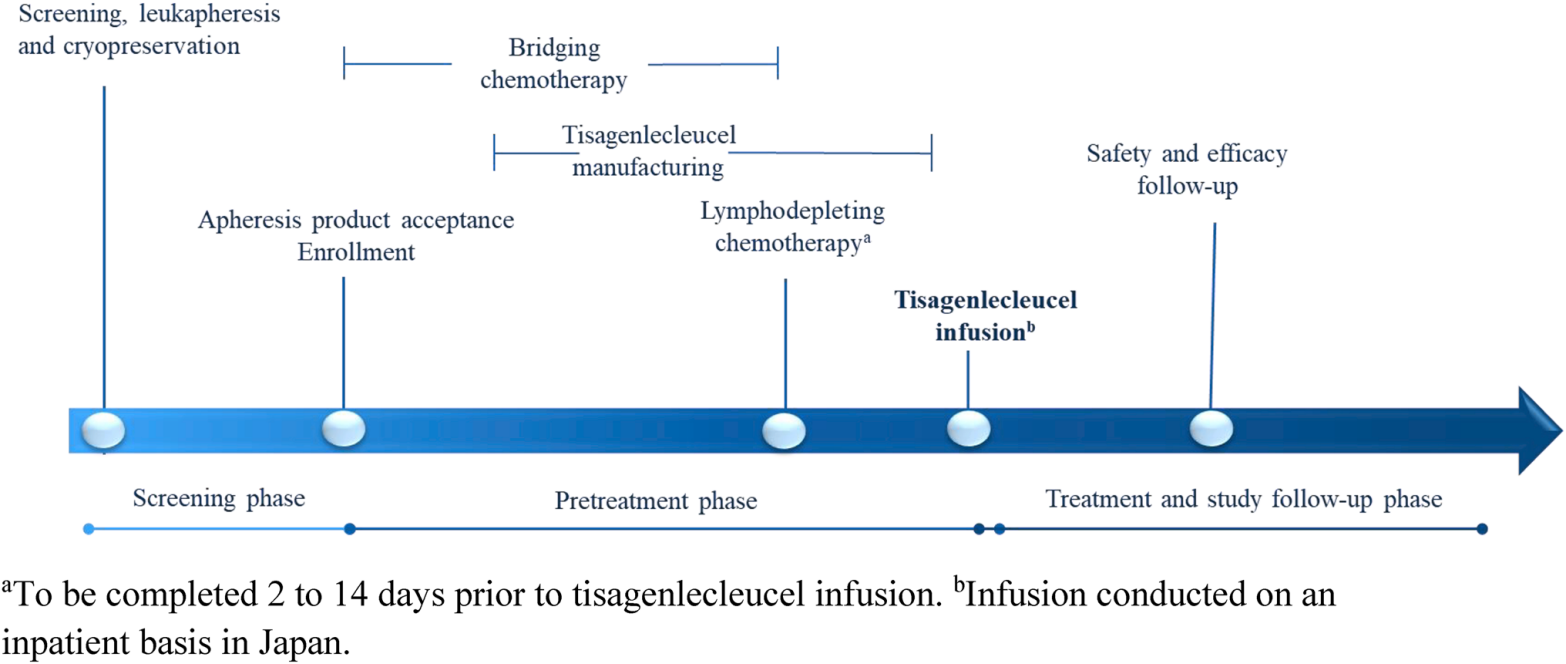
Study design

The design of the single-arm, open-label, phase 2, multicenter, global trial of tisagenlecleucel (JULIET) has been described previously (Fig. 1) [6]. Eligible patients were 18 years or older with r/r DLBCL who had previously received ≥ 2 lines of chemotherapy, including rituximab and anthracyclines. The current subgroup analysis included Japanese patients infused with tisagenlecleucel. Patients were excluded if they had previously received CD19-directed therapy or an allogeneic transplant or had primary mediastinal DLBCL or active central nervous system (CNS) involvement by DLBCL. Enrolled patients underwent bridging chemotherapy as needed and a round of lymphodepleting chemotherapy consisting of either fludarabine 25 mg/m^2^ and cyclophosphamide 250 mg/m^2^/day × 3 days or bendamustine 90 mg/m^2^/day × 2 days. Autologous leukapheresis products collected and cryopreserved at the clinical sites were shipped to the manufacturing facility (Morris Plains, NJ, USA) for tisagenlecleucel manufacturing, and the concentrated and cryopreserved cells were shipped back to the clinical sites for infusion. Patients received tisagenlecleucel infusion as a single target dose range of 1–5 × 10^8^ CD19-transduced viable CAR-T cells. The study was sponsored and designed by Novartis Pharmaceuticals Corporation and Novartis Pharma K.K. and was approved by the institutional review board at each participating institution. All patients provided written informed consent. Data were analyzed and interpreted by the sponsors in collaboration with all authors, and all authors reviewed the manuscript and vouch for the accuracy of the data and analysis.

### Endpoints

The primary endpoint was best ORR (CR + PR) per independent review committee [6], using the Lugano Classification [7] integrating anatomical imaging and functional imaging with positron emission tomography. Secondary endpoints included the duration of overall response (DOR), progression-free survival (PFS), event-free survival (EFS), OS, safety, and cellular kinetics.

### Statistical analysis

This is a subgroup analysis of all patients in the JULIET trial enrolled from medical institutions situated in Japan. Efficacy and safety analyses included all patients who received tisagenlecleucel infusion, and cellular kinetic analysis included all infused patients who had at least 1 sample providing evaluable cellular kinetic data for tisagenlecleucel. The current subgroup analysis was performed after all Japanese patients infused with tisagenlecleucel had completed at least 3 months of follow-up or were discontinued earlier (data cutoff date: May 21, 2018).

All data analyses were descriptive in nature owing to the small size of the Japanese subgroup. The primary efficacy endpoint of best ORR was reported with the exact binomial 95% confidence interval (CI). OS was illustrated by best overall response using a swimmer plot, along with relapse/progression status. For reporting of adverse events (AEs), the Medical Dictionary for Regulatory Activities (MedDRA), version 21.0, and the Common Terminology Criteria for Adverse Events (CTCAE), version 4.03 [8], were used. Specifically, the cytokine release syndrome (CRS) grading was reported according to the Penn grading scale [9]. Individual concentration–time profiles for tisagenlecleucel transgene levels assessed by quantitative polymerase chain reaction (qPCR) in peripheral blood were graphically presented by the month 3 disease response. A complete and detailed description of the overall study design and statistical analysis for the JULIET trial has been published previously [6].

## Results

**Fig. 2:**
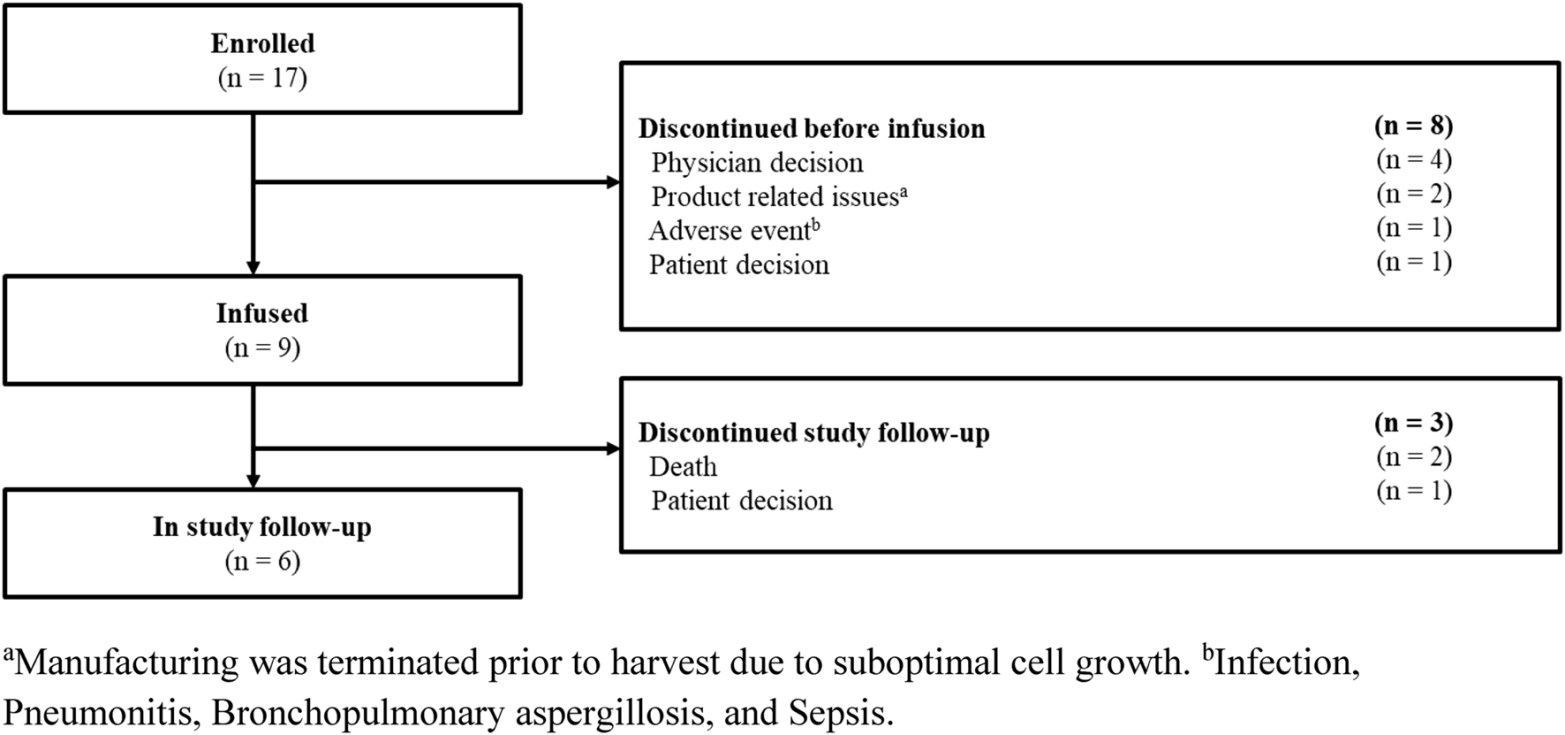
Patient disposition

Seventeen Japanese patients were enrolled, of which 9 were infused with tisagenlecleucel. Eight patients discontinued prior to tisagenlecleucel infusion due to disease progression (physician decision; *n* = 4), tisagenlecleucel product-related issues (*n* = 2), AEs (*n* = 1), and patient withdrawal (*n* = 1). Study follow-up was still ongoing for 6 patients infused with tisagenlecleucel. Three patients (2 deaths, 1 consent withdrawal) discontinued from the study follow-up (Fig. 2).

Of the 9 patients infused with tisagenlecleucel, 7 had relapsed disease and 2 had refractory disease at the start of the trial. The median age of the patients was 61 years, and 4 patients were aged more than 65 years. Four patients had received prior hematopoietic stem cell transplant. Prior lines of therapy ranged from 2 to 5. The histology review confirmed DLBCL in 7 patients, 1 of whom had transformed mucosa-associated lymphoid tissue lymphoma (Table 1). Bridging chemotherapy was used in six patients, and the treatment regimens included R-EPOCH, C-MOPP, R-ESHAP, R-GDP, and ICE. Overall disease responses prior to tisagenlecleucel infusion assessed by investigator were SDs in three patients, and CR, PR and progressive disease in one patient each. The median dose of tisagenlecleucel infusion was 2.0 × 10^8^ CAR-positive viable T cells (range, 1.0–4.9 × 10^8^ cells). The median time from enrollment to infusion was 63 days (range, 46–71).

**Table 1:** Patients’ demographics (infused patients, *N* = 9)

Baseline characteristics	Patient A	Patient B	Patient C	Patient D	Patient E	Patient F	Patient G	Patient H	Patient I
Age, years/sex	45/F	65/M	73/F	41/M	69/M	70/F	61/F	32/M	58/M
Prior HSCT	Yes	No	No	Yes	No	No	Yes	No	Yes
Histology review	DLBCL	Other^a^	DLBCL	DLBCL	DLBCL	tMALT lymphoma	DLBCL	DLBCL	DLBCL
Cell of origin^b^	ABC	NA	ABC	ABC	GCB	ABC	GCB	ABC	ABC
Previous lines of therapies	4	2	3	2	3	5	2	2	3
Disease status at the start of the trial	Relapsed	Relapsed	Relapsed	Relapsed	Relapsed	Refractory	Relapsed	Relapsed	Refractory
Double/triple hits	Negative	NA	Negative	Negative	Negative	Negative	Negative	*MYC* + *BCL6* + rearrangements	NA
Bridging chemotherapy prior to infusion	Yes	Yes	No	No	Yes	Yes	Yes	Yes	No
Investigator-assessed overall response prior to infusion	CR	SD	PD	PD	SD	PR	SD	PD	SD
LDH (U/L) prior to infusion^c^	144	331 H	222	264 H	497 H	202	261 H	510 H	354 H

*ABC* activated B cell, *DLBCL* diffuse large B-cell lymphoma, *F* female, *GCB* germinal center B cell, *HSCT* hematopoietic stem cell transplant, *LDH* lactate dehydrogenase, *M* male, *NA* not available*, tMALT* transformed mucosa-associated lymphoid tissue

^a^Neuroendocrine carcinoma

^b^Choi and modified Choi algorithms and Tally method

^c^H denotes a value above the upper limit of normal

### Efficacy

**Fig. 3:**
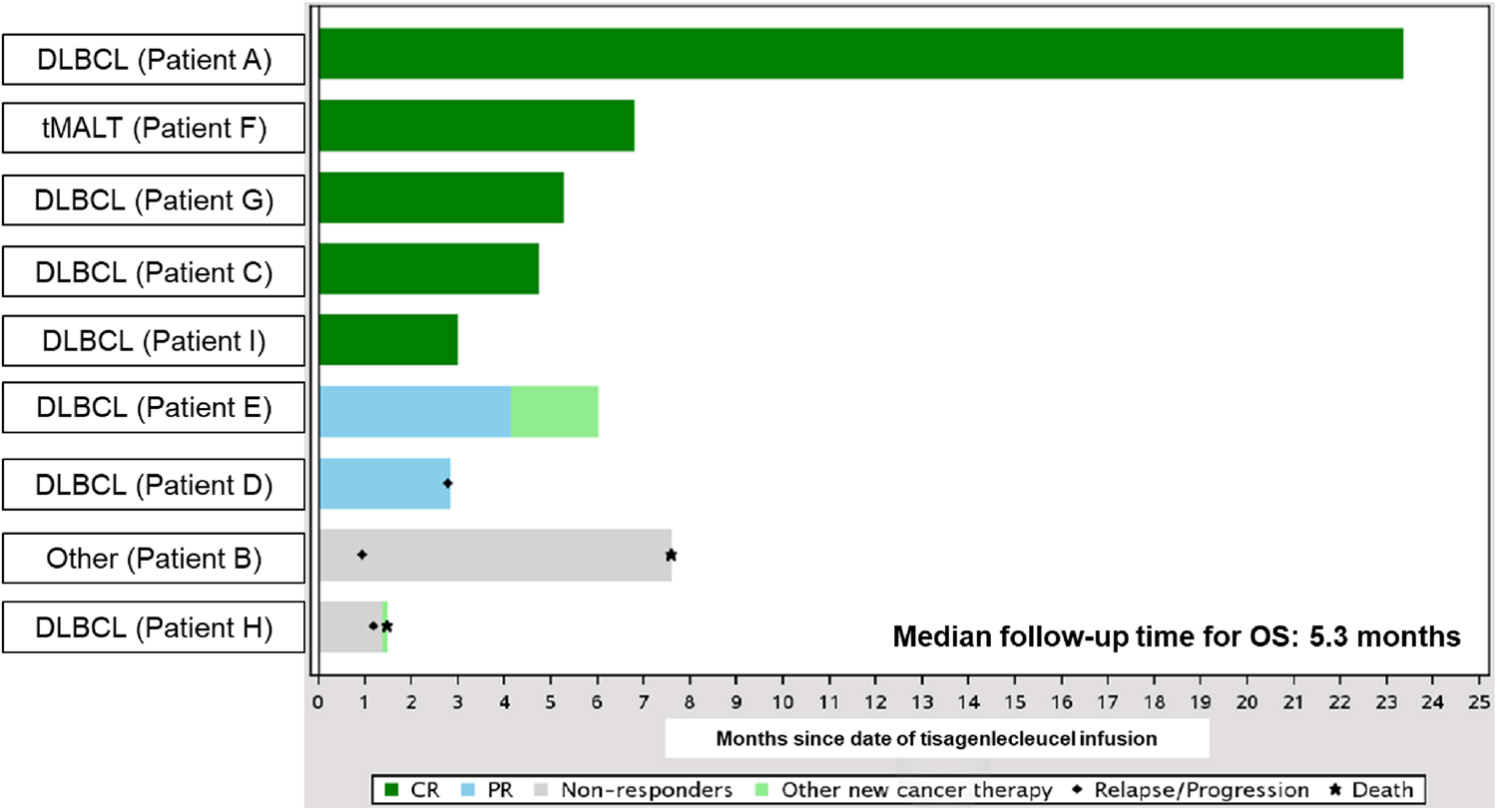
Overall survival with tisagenlecleucel by best overall response. *CR* complete response, *DLBCL* diffuse large B-cell lymphoma, *OS* overall survival, *PR* partial response, *tMALT* transformed mucosa-associated lymphoid tissue lymphoma

At data cutoff (May 21, 2018), the best ORR achieved was 77.8% (95% CI, 40.0–97.2), with 5 patients (55.6%) achieving a CR, 2 (22.2%) achieving a PR, and 2 (22.2%) with progressive disease (Table 2). At the month 3 follow-up, 5 patients (55.6%) were in CR and 1 (11.1%) patient had stable disease. Of the patients evaluated at the 6-month follow-up (*n* = 4), 2 patients were in CR. Among the 7 patients with a best response of CR or PR, the time to response ranged from 28 to 91 days, and the DOR ranged from 1 + to 523 + days. Among all infused patients, the PFS and EFS ranged from 29 to 550 + days, and the OS ranged from 45 to 710 + days (Table 2, Fig. 3). The DOR, PFS, EFS, and OS findings need to be interpreted with caution because the data for these endpoints were immature at the time of analysis, with considerably censored observations; therefore, the Kaplan–Meier estimates, such as median time, were not calculated for these endpoints.

**Table 2:** Overall efficacy and cellular kinetics (infused patients, *N* = 9)

Efficacy and cellular kinetic variables	Patient A	Patient B	Patient C	Patient D	Patient E	Patient F	Patient G	Patient H	Patient I
Best overall response	CR	PD	CR	PR	PR	CR	CR	PD	CR
Time to response, days	28	–	28	31	28	30	90	–	91
Duration of response, days	523+	–	51+	55	52+	148+	1+	–	1+
Progression-free survival, days	550+	29	78+	85	79+	177+	90+	36	91+
Event-free survival, days	550+	29	78+	85	126	207	90+	36	91+
Overall survival, days	710+	231	144+	86+	183+	207+	160+	45	91+
AUC_0–28d_, copies/µg × days	83,800	35,400	21,700	34,200	57,600	7290	189,000	1,740,000	2730
*C*_max_, copies/µg	9410	6120	2470	4420	5840	846	11,900	137,000	149
*T*_max_, days	5.82	5.85	8.78	12.9	5.73	6.96	11.0	26.7	19.8
*T*_1/2_, days	408	7.53	26.6	1.18	32.4	4.16	77.9	2.72	-

*AUC*_*0-28d*_ area under the concentration–time curve from time 0 to day 28, *C*_*max*_ maximum concentration, *CR* complete response, *PD* progressive disease, *PR* partial response, *T*_*1/2*_ half-life, *T*_*max*_ time to maximum expansion, + flags censored observations

### Safety

All 9 patients infused with tisagenlecleucel reported at least 1 AE of any grade severity within 8 weeks of tisagenlecleucel infusion. Grade 3 or grade 4 AEs reported in ≥ 15% of patients included CRS, anemia, decreased neutrophil count, decreased white blood cell count, decreased lymphocyte count, and decreased platelet count (Table 3). Among the AEs reported between 8 weeks and 1 year of infusion (*n* = 8), the most common (≥ 15% of patients) were nasopharyngitis and decreased white blood cell count, reported in 25% of patients each. The most common AEs of special interest (AESI) from any cause were cytopenias not resolved by day 28 (77.8%), CRS (66.7%), infections (44.4%), neurological events (11.1%), and tumor lysis syndrome (11.1%; Table 4). CRS was reported in 5/7 (71.4%) patients with disease response and 1/2 (50.0%) patients with no response. Grade 2 hypogammaglobulinemia was reported in one patient only, who received intravenous immunoglobulin (IVIG) four times from day 30 to day 149 (follow-up is ongoing). In total, four patients received IVIG after tisagenlecleucel infusion, which include three patients receiving IVIG since before tisagenlecleucel infusion. None of the four patients developed severe infection. Grade 3 and grade 4 cytopenias were reported in 6 patients (66.7%) and 1 patient (11.1%), respectively. In the patient who experienced grade 4 cytopenias, thrombocytopenia continued since before tisagenlecleucel infusion, worsened to grade 4 on day 19, and recovered to grade 3 on day 279 (after the data cutoff date). The patient received multiple platelet transfusions before and after tisagenlecleucel infusion. Grade 3 and grade 4 CRS were reported in 1 patient each (11.1%). Among the 6 patients who reported CRS, 3 patients required oxygen supplementation and 2 patients required anti-cytokine therapy with tocilizumab. The median time to onset of CRS was 4 days (range, 1–8; Table 5). Neurological event occurred in one patient, who developed grade 1 delirium on day 1, worsened to grade 3 on day 7 and resolved on the same day. Two deaths were reported due to disease progression more than 30 days after infusion: 1 patient died due to DLBCL and the other patient died due to his primary disease of neuroendocrine carcinoma.

**Table 3:** Adverse events ≥ 15%, reported within 8 weeks of infusion (infused patients, *N* = 9)

AEs (regardless of study drug relationship)	Patients (*N* = 9)		
	All grades, *n* (%)	Grade 3, *n* (%)	Grade 4, *n* (%)
Cytokine release syndrome	6 (66.7)	2 (22.2)	0
Anemia	4 (44.4)	2 (22.2)	0
Decreased neutrophil count	4 (44.4)	2 (22.2)	2 (22.2)
Decreased white blood cell count	4 (44.4)	2 (22.2)	1 (11.1)
Decreased lymphocyte count	3 (33.3)	1 (11.1)	2 (22.2)
Decreased platelet count	3 (33.3)	1 (11.1)	1 (11.1)
Decreased appetite	3 (33.3)	1 (11.1)	0

AEs are based on MedDRA preferred term

*AE* adverse event, *MedDRA* Medical Dictionary for Regulatory Activities

**Table 4:** Adverse events of special interest from any cause^§^ (infused patients, *N* = 9)

	Patients (*N* = 9)		
	All grades, *n* (%)	Grade 3, *n* (%)	Grade 4, *n* (%)
Cytokine release syndrome^a^	6 (66.7)	1 (11.1)	1 (11.1)
Neurological events^b^	1 (11.1)	1 (11.1)	0
Cytopenias not resolved by day 28	7 (77.8)	6 (66.7)	1 (11.1)
Infections	4 (44.4)	0	0
Tumor lysis syndrome	1 (11.1)	0	1 (11.1)

*AESI* adverse event of special interest, *MedDRA* Medical Dictionary for Regulatory Activities

^§^Occurring within 8 weeks of tisagenlecleucel infusion

^a^Cytokine release syndrome was graded using the Penn grading scale

^b^The neurological event reported in 1 patient was delirium. AESIs are based on grouped term consisting of MedDRA preferred term(s)

**Table 5:** Cytokine release syndrome

	Patients with CRS (*N* = 6)
Time to onset, median (range), days	4.0 (1–8)
Duration, median (range), days	7.5 (4–11)
Oxygen supplementation, *n* (%)	3 (50.0)
Anti-cytokine therapy, *n* (%)	2 (33.3)
Tocilizumab	2 (33.3)
Corticosteroids	0
ICU admission, *n* (%)	0^a^
Hypotension that required intervention, *n* (%)	0
High-dose vasopressors	0
Intubated, *n* (%)	0

*CRS* cytokine release syndrome, *HLH* hemophagocytic lymphohistiocytosis, *ICU* intensive care unit, *TLS* tumor lysis syndrome

^a^One patient, who developed CRS (day 8–11), HLH (day 10–42), and TLS (day 11–32), and admitted to the ICU (day 12–18), is not included

### Cellular kinetics

**Fig. 4:**
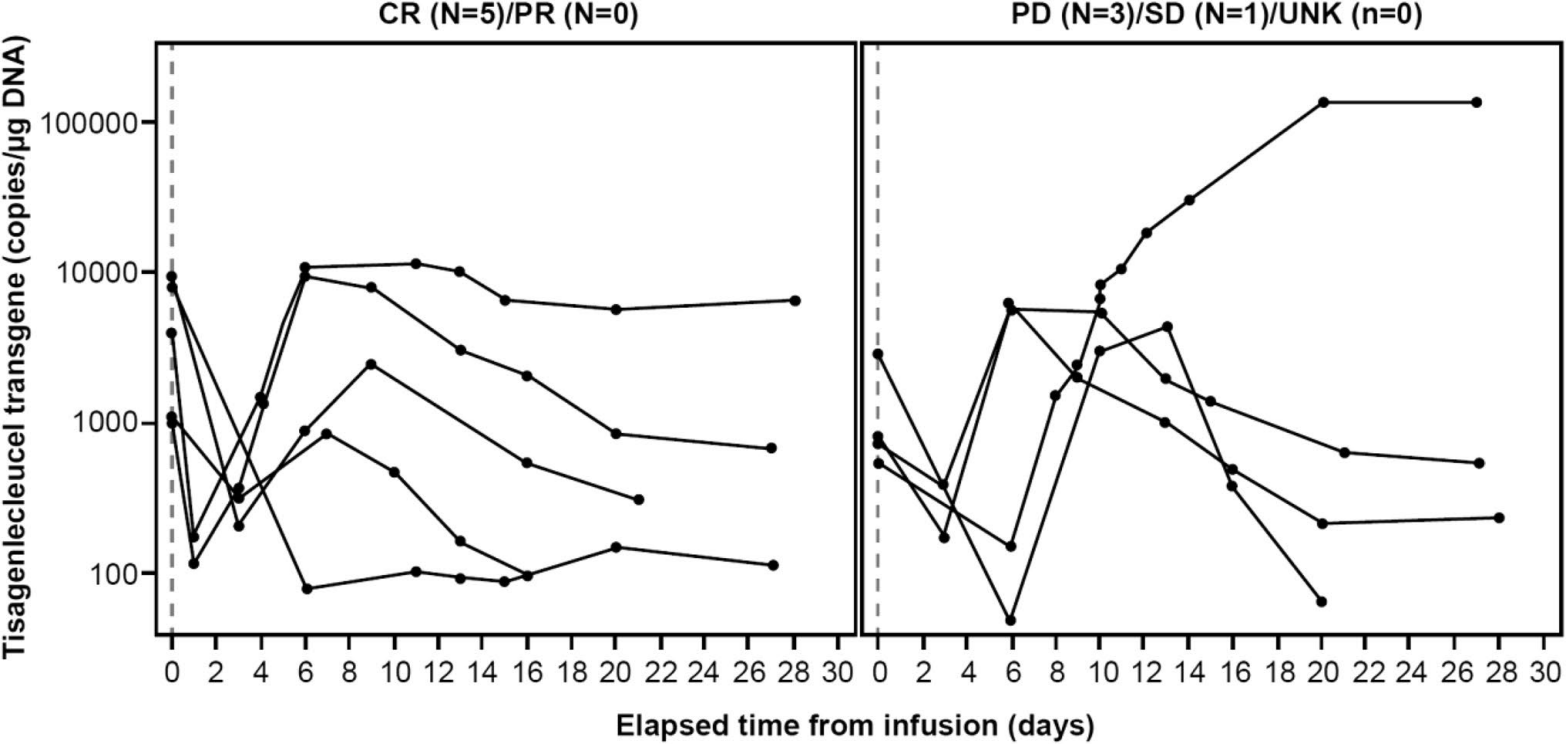
Tisagenlecleucel transgene levels assessed by qPCR up to month 1 in peripheral blood, by month 3 response as judged by the independent review committee. *CR* complete response, *DNA* deoxyribonucleic acid, *PD* progressive disease, *PR* partial response, *qPCR* quantitative polymerase chain reaction, *SD* stable disease, *UNK* unknown

Concentration–time profiles of tisagenlecleucel were generally similar between patients who had a month 3 response and those who did not (Fig. 4). Similar expansion, measured as maximum concentration (*C*_max_) and area under the concentration–time curve from time 0 to day 28 (AUC_0–28d_), was also observed in patients regardless of the response, with high variability in the transgene levels (Table 2). Thus, no apparent effect of exposure on clinical outcome was observed.

## Discussion

The JULIET trial is a phase 2, multicenter, global, pivotal trial of tisagenlecleucel, a second-generation anti-CD19 CAR T-cell therapy [6]. This subgroup analysis of JULIET assessed the efficacy, safety, and cellular kinetic profile of tisagenlecleucel in Japanese adult patients with r/r DLBCL. To our knowledge, this is the first report of an anti-CD19 CAR T-cell therapy in a Japanese subgroup to address the poor prognosis in this patient population. Tisagenlecleucel demonstrated substantial efficacy in selected Japanese patients with r/r DLBCL. The best ORR achieved was 77.8% (7/9; 95% CI, 40.0–97.2) with 5 CRs. Four out of 7 responders had an ongoing response at the time of data cutoff (median follow-up time for DOR and OS, 1.7 and 5.3 months, respectively), including 1 patient with a CR lasting for more than 17 months. One patient who had transformed mucosa-associated lymphoid tissue lymphoma achieved a CR with tisagenlecleucel. In the JULIET study (*n* = 93), tisagenlecleucel demonstrated durable responses, with a best ORR of 52%. The response rates at month 3 and month 6 were 38% and 33%, respectively [6]. Overall, efficacy was consistent between the overall and Japanese population. However, results from the Japanese subgroup should be interpreted with caution owing to the small number of patients.

Similar to tisagenlecleucel, high response rates with durable responses have also been observed with other anti-CD19 CAR T-cell therapies in the ZUMA-1 and TRANSCEND-NHL-001 trials [10, 11]. This confirms the role of anti-CD19 CAR T-cells for the treatment of r/r aggressive B-cell NHL. The overall JULIET study confirmed the durable ongoing responses in patients with r/r DLBCL who achieved a response (CR or PR) [6], suggesting possible sustained efficacy of tisagenlecleucel in the Japanese patients who achieved a response.

The relationships between several biomarkers and efficacy of tisagenlecleucel were assessed in the overall JULIET study, and multivariate analyses identified that the high levels of LDH, a known marker of tumor burden and disease aggressiveness, at pre-infusion were associated with poor efficacy outcomes [12]. In our subgroup analysis, two non-responders and four responders also had high LDH levels at pre-infusion (Table 1), showing that high levels of LDH at pre-infusion might not necessarily cause insufficient response in Japanese population.

In Japan, hospitalization for safety monitoring is required from initiation of lymphodepleting chemotherapy until 3 weeks after infusion, while patients could receive infusions in either inpatient or outpatient settings outside Japan. The safety profile of tisagenlecleucel in the Japanese patients was consistent with that in the overall population (CRS, 58%) [6]. CRS was reported in 67% of the Japanese patients. All CRS was resolved within two weeks and was manageable with appropriately trained study-site personnel. No deaths were attributed to tisagenlecleucel, CRS, or cerebral edema. The 2 deaths reported in the Japanese subgroup occurred due to disease progression more than 30 days after tisagenlecleucel infusion. In the JULIET trial, CRS was graded using the Penn grading scale and was managed with tocilizumab as per protocol-specific algorithm [6], which included administration of anti-cytokine therapy for patients who did not respond to supportive care. The cellular kinetics of tisagenlecleucel observed in the Japanese subgroup were consistent with that observed in the overall population despite high variability [6]. Eight patients did not receive infusion mainly due to physician decision of disease progression in 4 patients after leukapheresis, suggesting the importance of disease control during the manufacturing process and patient selection. The dropout rate in Japanese patients is similar to that in the overall JULIET study, and the high dropout rate may be associated with a long turn-around time. It might be an important challenge in the future to shorten the turn-around time of CAR T-cell therapy to improve the accessibility of CAR T-cell therapy in Japan. Bridging chemotherapy was used to maintain optimal health during the manufacturing process as needed. Before tisagenlecleucel infusion, overall disease response was assessed by investigator, and CR was achieved in one patient. After tisagenlecleucel infusion, the response continued more than 523 days, which highlights the importance of further investigation on optimal bridging chemotherapy for achieving durable response.

There are two other Japanese patients to be highlighted. Manufactured product for one patient did not meet a release criterion of cell viability, which is out of specification (OOS). The OOS product was provided to the patient through an exceptional release procedure. The procedure includes assessment of a product quality, efficacy and safety risk performed by the study sponsor, followed by assessment of risk/benefit by the investigator, confirming that the use of OOS product provides a potential patient benefit that outweighs the disease to be treated with the OOS product. Informed consent for the release of OOS product was obtained from the patient. No severe AE was reported after tisagenlecleucel infusion, and CR was achieved. However, the appropriateness of providing OOS product should be evaluated on a case-by-case basis in the future studies. One patient with DLBCL fulfilling the enrollment criteria was enrolled into this study but had a relapse in lung several months prior to the study entry. This relapse was retrospectively diagnosed as neuroendocrine carcinoma a few months after tisagenlecleucel infusion. It was not clinically feasible to perform biopsy on this patient prior to enrollment and this deviation may represent a challenge in selecting patients for tisagenlecleucel treatment in clinical practice. The present subgroup analysis has several limitations, including the limited number of patients; a high rate of dropouts after enrollment, which may be associated with a long turn-around time; and a relatively short follow-up duration. To confirm the actual role of tisagenlecleucel in Japan, further evaluation in a larger number of Japanese patients with a sufficient follow-up duration is required.

Despite several limitations, the results of this subgroup analysis support the use of tisagenlecleucel in Japanese patients with r/r DLBCL who have no effective treatment options. The high response rate and manageable safety profile observed with tisagenlecleucel are promising and offer a potential new treatment option for Japanese adult patients with r/r CD19-positive DLBCL.
